# High Incidences of Invasive Fungal Infections in Acute Myeloid Leukemia Patients Receiving Induction Chemotherapy without Systemic Antifungal Prophylaxis: A Prospective Observational Study in Taiwan

**DOI:** 10.1371/journal.pone.0128410

**Published:** 2015-06-10

**Authors:** Jih-Luh Tang, Hsiang-Chi Kung, Weng-Chi Lei, Ming Yao, Un-In Wu, Szu-Chun Hsu, Chien-Ting Lin, Chi-Cheng Li, Shang-Ju Wu, Hsin-An Hou, Wen-Chien Chou, Shang-Yi Huang, Woei Tsay, Yao-Chang Chen, Yee-Chun Chen, Shan-Chwen Chang, Bor-Sheng Ko, Hwei-Fang Tien

**Affiliations:** 1 Division of Hematology, Department of Internal Medicine, National Taiwan University, Taipei, Taiwan; 2 Tai-Cheng Stem Cell Therapy Center, National Taiwan University, Taipei, Taiwan; 3 Division of Infection, Department of Internal Medicine, National Taiwan University, Taipei, Taiwan; 4 Clinical Trial Center, National Taiwan University Hospital, Taipei, Taiwan; 5 Department of Laboratory Medicine, National Taiwan University Hospital, Taipei, Taiwan; 6 National Institute of Infectious Diseases and Vaccinology, National health Research Institute, Miaoli, Taiwan; The University of Hong Kong, HONG KONG

## Abstract

Invasive fungal infections (IFIs) is an important complication for acute myeloid leukemia (AML) patients receiving induction chemotherapy. However, the epidemiological information is not clear in Southeastern Asia, an area of potential high incidences of IFIs. To clarify it, we enrolled 298 non-M3 adult AML patients receiving induction chemotherapy without systemic anti-fungal prophylaxis from Jan 2004 to Dec 2009, when we applied a prospective diagnostic and treatment algorithm for IFIs. Their demographic parameters, IFI characters, and treatment outcome were collected for analysis. The median age of these patients was 51 years. Standard induction chemotherapy was used for 246 (82.6%) patients, and 66.8% of patients achieved complete remission (CR) or partial remission. The incidence of all-category IFIs was 34.6% (5.7% proven IFIs, 5.0% probable IFIs and 23.8% possible IFIs). *Candida tropicalis* was the leading pathogen among yeast, and lower respiratory tract was the most common site for IFIs (75.4%, 80/106). Standard induction chemotherapy and failure to CR were identified as risk factors for IFIs. The presence of IFI in induction independently predicted worse survival (hazard ratio 1.536 (1.100–2.141), *p* value = 0.012). Even in those who survived from the initial IFI insults after 3 months, the presence of IFIs in induction still predicted a poor long-term survival. This study confirms high incidences of IFIs in Southeastern Asia, and illustrates potential risk factors; poor short-term and long-term outcomes are also demonstrated. This epidemiological information will provide useful perspectives for anti-fungal prophylaxis and treatment for AML patients during induction, so that best chances of cure and survival can be provided.

## Introduction

Although the control of bacterial infections in patients with hematological malignancies has been significantly improved with broad-spectrum antibiotics in the past decades, treating invasive fungal infections (IFI) is still a major problem in these patients, especially in patients with prolonged neutropenia after chemotherapy. Epidemiological data from previous studies have shown that the incidence of IFIs in patients with hematologic malignancies has increased dramatically in the past years[1], substantially increasing morbidity and mortality rates. In addition, over half of IFIs emerge during the remission induction chemotherapy[2]. Several factors have been identified that negatively influence the outcome of IFI, including old age, use of corticosteroid, an absolute neutrophil count (ANC) of less than 0.1X10^9^/L at the time of IFI diagnosis, lack of recovery from aplasia and multiple pulmonary localizations of infection[3].

Incidences in different countries of IFI in hematologic malignancies have been previously demonstrated[2,4–12], mainly from countries located in temperate zones. The epidemiology of IFI in patients with hematological malignancies in subtropic or tropic regions should be different due to favorable fungal growth conditions, but to date, there is no convincing data available for patients in these regions. Furthermore, other factors such as the genetic background of patients, chemotherapeutic regimens or environmental settings also contribute to the geographic variation in IFI epidemiology of these patients[13]. This study is therefore aimed at providing informative epidemiologic results about IFI in acute myeloid leukemia (AML) patients receiving induction chemotherapy in Taiwan. Furthermore, we will illustrate the potential risk factors for IFIs, and also the potential short-term or long-term prognostic impacts of IFIs on the survival of these patients. These results should provide useful perspectives in establishing guidelines for anti-fungal prophylaxis in Southeastern Asia, and also treatment in patients with hematological malignancies.

## Patients and Methods

### Hospital setting, patient population and data collection

This observational study was conducted as a part of a hospital-wide active and prospective surveillance of healthcare-associated infection program[14] at the National Taiwan University Hospital, which is a 2300-bed teaching hospital providing primary and tertiary care in Northern Taiwan. All newly diagnosed non-M3 AML adult patients (aged more than 16) hospitalized from January 1, 2004 to December 31, 2009 for chemotherapy were enrolled in this study. They were managed according to the standard of care and were followed by at least one of the investigators. Data were collected then by chart reviews and included the following parameters: age, gender, antecedent hematologic disease, cytogenetic results at diagnosis, induction regimens, treatment response, laboratory findings such as imaging, histopathology and fungal antigen assay (galactomannan antigen and cryptococcal antigen assay) and fungal cultures, treatment outcome of IFIs and mortality.

### Ethics information

This observational study was approved by the Research Ethic Committee of National Taiwan University Hospital, and the policy that informed consents can be waived for this analysis was also approved by the Research Ethic Committee because the data were analyzed anonymously.

### Treatment and response criteria of AML

Standard induction chemotherapy for non-M3 AML in this study was idarubicin + cytarabine (idarubicin 12mg/m^2^ per day for 2–3 days, and cytarabine 100mg/m^2^/day for 5–7 days). Other chemotherapy regimens with less intensity were classified as low-intensity regimens. During induction chemotherapy, no routine prophylactic granulocyte colony-stimulating factor (G-CSF) was used. They were only applied in cases of poor performance status and upon the physicians’ decisions.

The definition of treatment response generally followed the European Leukemia network (ELN) 2010 recommendation[15], but was simplified: complete remission (CR) was defined as a blast count of <5% in the bone marrow; partial remission (PR) was defined as a decrease in bone marrow blasts by 50% but still remaining in a range of 5 to 25%; resistant disease (RD) was referred to those who failed to achieve CR or PR in bone marrow examination after chemotherapy, and “undefined” response was referred to those who had no available results of bone marrow examination after chemotherapy. Survival was measured from the first day of induction chemotherapy (Day 0) to the date of expiration or the date of the last follow-up. Induction mortality was defined as all-cause-mortality within 42 days following induction chemotherapy or till next cycle of chemotherapy. Mortality attributed to IFI was defined as death with continuing symptoms and/or signs of IFI.

### Antimicrobial prophylaxis and infection control measures

Patients received prophylactic treatment during the neutropenic period following chemotherapy consisting of sulfamethoxazole (400mg/trimethoprim 80mg once or twice daily), and oral nystatin suspension (for oropharyngeal candidiasis). They did not receive any other systemic antibacterial, antifungal or antiviral prophylactic treatment, and were not kept in positive-pressure protective room with a HEPA-filter. Routine bacterial and fungal surveillance was conducted for patients with prior prolonged hospitalization (more than one month). For those who developed IFI, environment fungal surveillance was performed.

### Diagnosis, definition and treatment of invasive fungal infection

The European Organization for Research and Treatment of Cancer/Mycosis Study Group (EORTC/MSG) 2008 consensus criteria was used to define IFIs in this study[16], and IFIs were categorized as proven, probable or possible. Only IFIs appearing before the next cycle of chemotherapy were counted; if no next cycle of chemotherapy was given, the IFIs were diagnosed no later than day +42 after the induction or chemotherapy. Mucocutaneous fungal infections were not included in this study. In order to improve the accuracy in classifying IFIs, possible IFIs were followed for 60 days to exclude the possibility of tuberculosis or other non-infectious etiologies and also to assess the response to antifungal agents[17].

During the study period, a diagnostic and treatment algorithm for patients with hematological malignancies was applied for evaluating patients with IFI risks. The algorithm was basically designed with a diagnosis-driven strategy, as follows: When patients remain febrile >72hrs after broad-spectrum anti-bacterial agents in neutropenia, a thorough history and physical evaluation will be taken, along with cultures for blood and other potential infection focuses. For patients with no identified infection focus, high-resolution computed tomography (HRCT) will be performed, and a Platelia *Aspergillus* galactomannan enzyme-linked immunoassay[18] (ELISA) (Bio-Rad Laboratories, Hercules, CA) will be performed twice weekly. Broncho-alveolar lavage (BAL) was not routinely performed in these patients. If any specific infection focus was noted on the HRCT or in other surveillance studies and the general conditions of the patient were suitable, invasive studies to obtain tissues for mycological cultures or pathological examination were performed as soon as possible.

Preemptive anti-fungal agents, such as echinocandins, fluconazole or voriconazole, were given if IFIs were suspected with the HRCT findings, positive galactomannan assays, or other evidences. If IFIs were still highly suspected but no clinical evidence was available, empirical antifungal agents such as amphotericin-B (1–1.5mg/Kg/day intravenously) or caspofungin (50mg/day intravenously) were given upon the physicians’ approval. After anti-fungal agents were given, evaluation of the treatment efficacies, clinical evidences of IFIs and adverse effects related to drugs were done every 3–4 days, or as needed.

### Statistical analysis

In recording the survival time, those patients who received hematopoietic stem cell transplantation were censored on the day of stem cell infusion. Fisher’s exact test, chi-square test and the Mann-Whitney test were used to identify the statistical differences when appropriate. Kaplan-Meier analysis and a log-rank test were used to compare overall survival (OS) between different groups of patients. Cox proportional hazards regression models were applied for multivariate analysis to find the variables influencing overall survival. A two-sided *p* value of less than 0.05 was denoted as statistically significant. All the statistical analysis was performed with SPSS software version 17.0 (SPSS Inc, Chicago, IL).

## Results

### Patient characteristics

From Jan 2004 to Dec 2009, 298 adult patients with newly diagnosed non-M3 AML received induction chemotherapy and were enrolled. The median follow-up time for survival was 16.6 month (ranging from 0.1 to 74.1 months), and the overall time subjected to the evaluation for IFIs after induction chemotherapy was 371.1 patient-months.

Their demographic characteristics are shown in Table 1. The median age was 51 years (range, 16–87 years), with a male-to-female ratio of 1.13:1. Antecedent hematologic diseases were noted in 19.1% (57/298) of the patients, including 34 with myelodysplastic syndrome (MDS) and 18 with myeloproliferative neoplasms (MPNs). Forty-six (15.4%) patients were classified into the favorable-cytogenetic group, 52 (17.4%) into unfavorable group, and 191 (64.1%) into intermediate risk group. Standard induction chemotherapy was given in 82.6% (246/298) of the patients, and low-intensity therapy in 52 (17.4%) of them. After 1^st^ induction therapy, 161 (54.0%) achieved CR, and 38 (12.8%) achieved PR. Twenty-night (9.7%) induction chemotherapy accompanied with mortality.

**Table 1:** Clinical characteristics of enrolled patients.

Characters	Number (%)
All patients	298 (100.0%)
Age, years	
Median [range]	51 [16–87]
Gender	
Male/Female [ratio]	158/140 [1.13:1]
Antecedent hematologic malignancy	
Myelodysplastic syndrome	34 (11.4%)
Myeloproliferative neoplasms	18 (6.0%)
Others*	5 (1.7%)
None	241 (80.9%)
Cytogenetic risk	
Favorable	46 (15.4%)
Intermediate	191 (64.1%)
Unfavorable	52 (17.4%)
Not available	9 (3.0%)
Induction regimen	
Standard	246 (82.6%)
Low-intensity	52 (17.4%)
Response of induction chemotherapy	
Complete remission	161 (54.0%)
Partial remission	38 (12.8%)
Resistant disease	76 (25.5%)
Undefined response	23 (7.7%)
Induction mortality (within 42 days)	
Yes	29 (9.7%)
No	269 (90.3%)

*Including 1 severe aplastic anemia, 1 non-Hodgkin lymphoma, 1 paroxysmal nocturnal hemoglobinuria and 2 myelofibrosis

### Incidence, causative pathogens, and infection sites of invasive fungal infection

In the 298 courses of induction chemotherapy, 103 (34.6%) all-category IFIs were identified, including 17 (5.7%) proven, 15 (5.0%) probable and 71 (23.9%) possible IFIs. The incidence density for proven, probable and possible IFIs was 0.046, 0.040 and 0.191 per patient-month, respectively.

Table 2 shows the distribution of fungal pathogens and methods of microbiological diagnosis. Of the 34 etiological pathogens identified in 32 patients with proven or probable IFIs, 21 (61.8%) were mold, including 13 that were exclusively diagnosed with two positive galactomannan tests. Among the remaining 21 IFIs with culture and/or histopathology evidences, yeasts were more common than mold (13 vs. 8). *Candida tropicalis* was the most common yeast identified (4 cases), followed by 3 *Candida albicans* and 2 *Candida parapsilosis* isolates.

**Table 2:** Identified 34 etiological pathogens* in 32 IFI cases.

Fungal species	Number (%)
Overall	34 (100.0%)
Yeast	13 (38.2%)
*Candida spp*.	12
*Candida tropicalis*	4
*Candida albicans*	3
*Candida parapsilosis*	2
Not specified^#^	3
*Rhodotorula*	1
Mold	21 (61.8%)
Positive GM tests only	13
*Aspergillus spp*.	5
*Aspergillus fumigates*	1
Not specified^&^	4
*Fusarium*	1
*Mucormyces*	1
*Mycelium sterile*	1

*Two cases had more than one species of fungal infection

^#^ Including cases with yeasts identified in tissue but negative cultures

^&^Including cases with hyphae identified in tissue but negative cultures

GM, galactomannan

The infection sites of all categories of IFI were also analyzed. Of all the 106 infection sites identified in 103 patients with IFIs, the most common location of infection was the lower respiratory tracts (75.4%, 80/106), followed by blood stream infections only (9.4%, 10/106), hepatosplenic microabscesses (6.6%, 7/106), upper aero-digestive tracts (3.8%, 4/106) and 5 other sites (1 genito-urinary tracts, 2 skin, 1 intestines and 1 central nervous system).

### Risk factors associated with invasive fungal infection

The correlation between the incidences of IFIs and patient characteristics is illustrated in Table 3. There was no statistical correlation with the IFI incidences to gender. Those who aged 40–59 years old had less incidence of all-categories of IFIs than those are younger or older, but the incidences of proven/probable IFIs were similar in all the 3 age groups. Acute leukemia patients with known antecedent hematological diseases had more IFIs than those without (45.6% vs. 32.0%) for all categories of IFIs, but not for proven/probable IFIs. The response to chemotherapy was also correlated with the incidences of IFIs. Those who were resistant to the first cycle of induction or chemotherapy had a statistically higher chance to develop IFIs (48.7% for all-category IFIs or 14.5% for proven/probable IFIs). Interestingly, patients who received standard chemotherapy seemed to have no increased risk for all-category IFIs. However, when only proven or probably IFIs were considered, the risk significantly increased in comparison with those who received only low-intensity chemotherapy (12.6% vs 1.9%, *p* value = 0.013).

**Table 3:** Univariate analysis for risk factors associated with IFIs among AML patients with induction chemotherapy.

	With proven or probable IFIs	*p* value	With all-category IFIs	*p* value
	n (%)		n (%)	
All patients (N = 298)	32 (10.7%)		103 (34.6%)	
Age		NS		0.046
≧60 years (n = 91)	12 (13.2%)		35 (38.5%)	
40–59 years (n = 121)	11 (9.1%)		32 (26.4%)	
16–39 years (n = 86)	9 (10.5%)		36 (41.9%)	
Sex		NS		NS
Male (n = 158)	19 (12.0%)		60 (38.0%)	
Female (n = 140)	13 (9.3%)		43 (30.7%)	
Antecedent hematologic disease		NS		0.038
Yes (n = 57)	8 (14.0%)		26 (45.6%)	
No (n = 241)	24 (10.0%)		77 (32.0%)	
Chemotherapy		0.013		NS
Standard (n = 246)	31 (12.6%)		87 (35.4%)	
Low-intensity (n = 52)	1 (1.9%)		16 (30.7%)	
Response to chemotherapy		0.046		0.039
CR (n = 161)	13 (8.1%)		43 (26.7%)	
PR (n = 38)	3 (7.9%)		10 (26.3%)	
RD (n = 76)	11 (14.5%)		37 (48.7%)	
Undefined (n = 23)				

AML, acute myeloid leukemia; CR, complete remission; IFI, invasive fungal infection; PR, partial remission; RD, resistant disease

When multivariate analysis was encountered for identifying potential factors predicting IFIs after induction for non-M3 adult AML, we found that standard induction chemotherapy and failure to achieving CR were the significant risk factors for all categories of IFIs, and those who aged between 40 to 59 years old had a trend of less infection risk. (as shown in Table 4). But if only proven/probable IFIs were considered, only standard induction chemotherapy was related to higher risk.

**Table 4:** Multivariate analysis for risk factors associated with IFIs among AML patients with induction chemotherapy.

	With proven or probable IFIs		With all-category IFIs	
	Estimated hazard ratio	*p* value	Estimated hazard ratio	*p* value
	(95% CI)		(95% CI)	
Antecedent hematological diseases (Yes vs No)	1.404 (0.563–3.509)	0.466	1.610 (0.864–2.994)	0.134
Age		0.151		0.096
≧60 vs. 16–39 years	2.105 (0.803–5.517)	0.130	0.972 (0.503–1.878)	0.932
40–59 vs. 16–39 years	0.897 (0.349–2.308)	0.822	0.511 (0.227–1.083)	0.080
Sex (Male vs. Female)	1.449 (0.665–3.158)	0.351	1.419 (0.858–2.346)	0.173
Induction chemotherapy (Standard vs Low-intensity)	15.345 (1.911–123.20)	0.010	1.663 (1.030–3.195)	0.041
Response to chemotherapy (CR vs non-CR)	0.528(0.238–1.171)	0.116	0.494 (0.270–0.904)	0.022

AML, acute myeloid leukemia; CI, confidence interval; CR, complete remission; IFI, invasive fungal infection

### Adverse survival impact of invasive fungal infection

Within 42 days of induction chemotherapy, 29 (9.7%) patients died. Twenty deaths were attributed to IFIs. accounting for 68.9% (20/29) of these early deaths. The overall IFI-attributed mortality during induction chemotherapy (within 42 days) was 6.7% (20/298) for all patients and 19.4% (20/103) for patients with any categories of IFIs.

**Fig 1:**
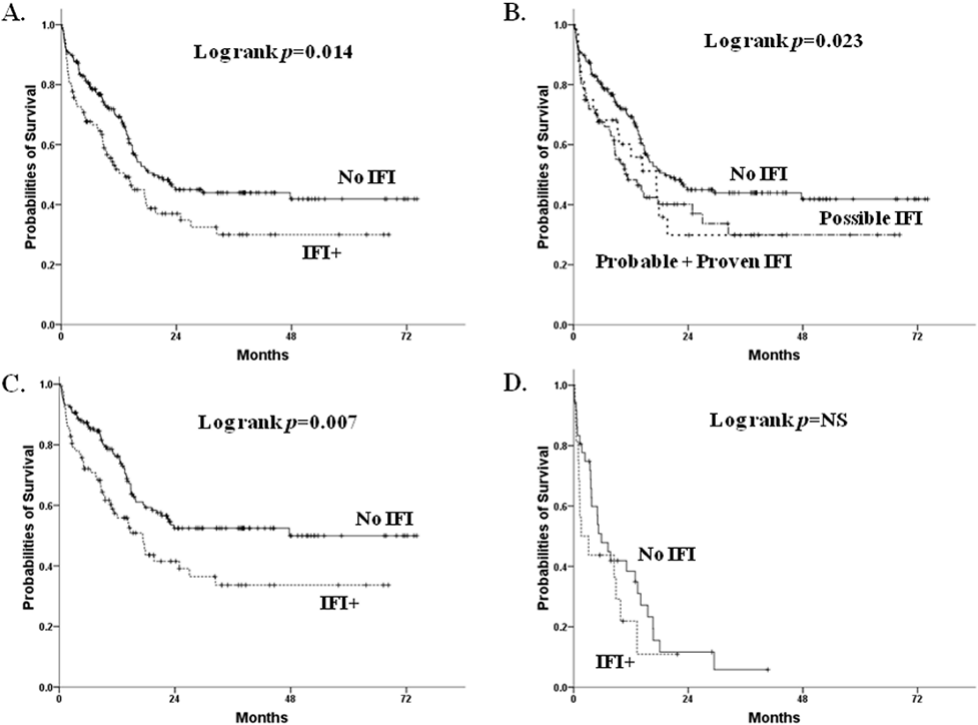
Kaplan-Meier curves for AML patients receiving induction chemotherapy, according to IFI status. (A) For all patients with or without any IFIs. (B) For all patients with different categories of IFIs. (C) For patients receiving standard induction chemotherapy. (D) For patients receiving low-intensity regimens as induction chemotherapy.

Non-M3 AML patients with all-category IFIs during induction would have shorter median survival than those without any IFIs (13.3±2.4 vs. 19.1±3.1 months, log rank *p* value = 0.014) (Fig 1A). No matter which IFI category was diagnosed, the median survival for these patients was uniformly shorter than those without IFIs (proven/probable vs. no IFIs, 17.3±2.6 vs. 19.1±3.1 months, log rank *p* value = 0.053; possible vs. no IFIs, 11.3±3.1 vs. 19.1±3.1 months, log rank p = 0.013; overall log rank *p* value = 0.023) (Fig 1B). However, the median survival was not significantly different between different categories of IFIs. In patients receiving standard induction chemotherapy, survival difference between those with and without all-category IFIs was significant (17.3±2.8 months vs. not reached, log rank *p* value = 0.007, as in Fig 1C); but in those with low-intensity chemotherapy, the survival difference was not apparent (1.6±1.8 vs. 5.8±1.6 months, log rank *p* value not significant, as in Fig 1D). A further multivariate analysis with Cox proportional hazards regression models revealed that the presence of IFIs serves as a poor prognostic marker for survival, which is independent of other risk factors such as cytogenetics, age and induction treatment (Table 5).

**Table 5:** Cox-proportional hazards survival analysis for AML patients with induction chemotherapy.

Variables	Estimated hazard ratio	*p* value
	(95% CI)	
Antecedent hematological disease (Yes vs. No)	0.982 (0.653–1.477)	0.930
Age		
≧60 vs. 16–39 years	1.959 (1.266–3.301)	0.003
40–59 vs. 16–39 years	1.644 (1.068–2.532)	0.024
Induction chemotherapy (Standard vs Low-intensity)	0.446 (0.256–0.665)	<0.001
Cytogenetic risk (Favorable vs. Others)	0.261 (0.178–0.381)	<0.001
Invasive fungal infection* (Yes vs. No)	1.637 (1.159–2.309)	0.005

*Indicating proven, probable and possible cases.

AML, acute myeloid leukemia; CI, confidence interval; CR, complete remission

**Fig 2:**
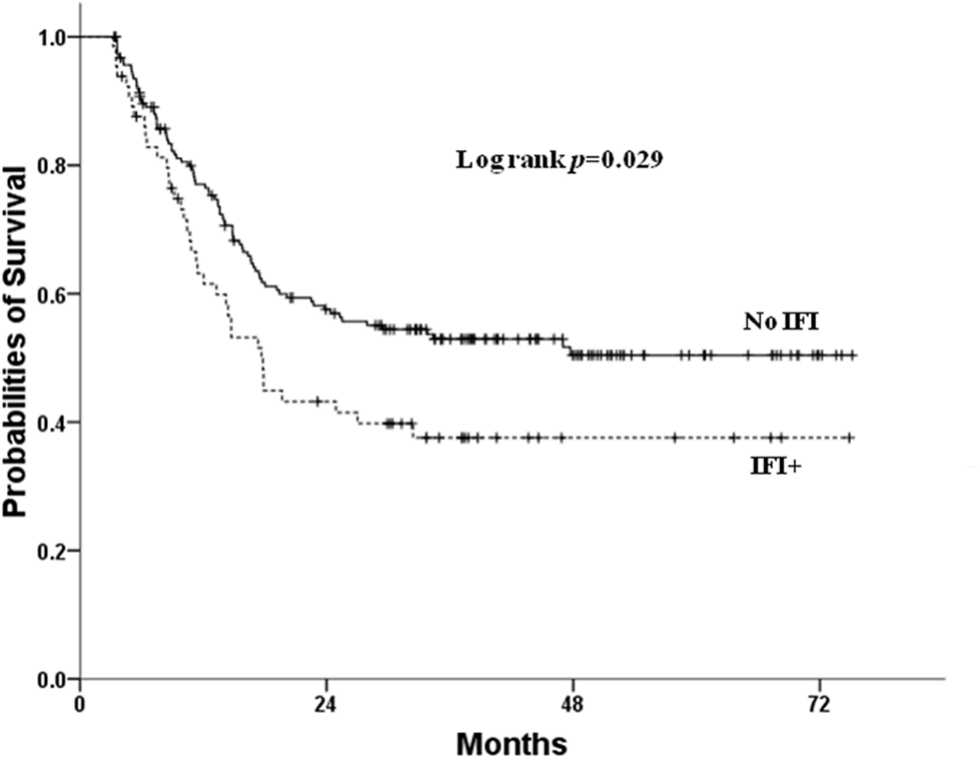
Kaplan-Meier curves for 3-month survivors after induction chemotherapy, according to IFI status.

To assess the long-term survival impacts of IFIs, we analyzed the outcome for 250 patients who survived for more than 3 months after chemotherapy, in whom the effects of early IFI-attributed deaths were ameliorated. As shown in Fig 2, the presence of IFIs still predicts a significantly worse median survival time (not reached vs. 17.7±1.9, log rank *p* value = 0.029).

## Discussion

This observational study is the first population-based epidemiological report about the incidences of IFIs in AML patients receiving induction chemotherapy in Southeastern Asia with humid and warm climates. In addition, this report is among those with enough follow-up time to demonstrate both the short-term and long-term impacts of IFIs on the outcomes of AML. Furthermore, in a prospective manner, this study shows the usefulness of surveillance results using an algorithm based diagnostic-driven strategy to manage IFIs in these patients. Furthermore, it is especially worthy to note that no routine anti-fungal prophylaxis was given to the patients in the study population. We also analyzed in detail the predicting factors for IFI development and the short-term and long-term prognostic impacts of IFIs on survival of patients. These results are all critical for developing therapeutic strategies and for designing interventional clinical trials with similar diagnostic and treatment approaches in this area in the future. The high incidence of IFIs revealed by this report should also raise different cost-effectiveness consideration about the requirement of anti-fungal prophylaxis in AML patients with induction chemotherapy, especially in areas with proper environment factors for the growth of fungi.

We reported that the incidences of IFIs are 34.6% for all-category IFIs, and 10.7% for proven or probable IFIs in acute leukemia patients receiving induction chemotherapy. The incidences are among the highest in comparison with other recent reports with large-scaled retrospective cohorts, and even higher than the latest report from a prospective cohort in Italy by Caira et al (as summarized in Table 6)[4,6,8–12]. The reported incidences greatly varied, from 4.0% to 48.4%, indicating significant varieties in patient populations, chemotherapy regimens, anti-fungal prophylaxis, geographic variation and other factors. The high incidence in our report can be substantially explained by several factors, including the lack of routinely administered anti-fungal prophylaxis, and a climate of high temperature and humidity, which facilitates the growth of fungi. Other environmental factors such as water contamination may contribute to fungal growth as well. The impacts of environmental factors on IFI incidences can be substantially illustrated in the report by Caira et al.[12], in which house renovation, types of job, types of habits and cigarette/cocaine use are proven to be the risk factors of IFIs during induction chemotherapy for AML. Although the follow-up time frame is different from our study, Nucci et al. also report a high 1-yr incidence of 18.7% for proven/probable IFIs in AML patients after diagnosis in Brazil[19], a place with climates similar to Taiwan.

**Table 6:** Recent large-scaled epidemiological studies for invasive fungal infections in adult patients with hematological malignancies.

	Gomes, et al	Ananda-Rajah, et al	^$^Malagola, et al	Hammond, et al	Neofytos, et al	Kurosawa, et al	Caira, et al	Current series
	2014[6]	2012[4]	2008[10]	2010[8]	2013[11]	2012[9]	2015[12]	
**Regions**	US	Australia	Italy	US	US	Japan (Hokkaido)	Italy	Taiwan
**Year**	2009–2011	1998–2010	1997–2002	2004–2006	2005–2010	2006–2008	2010–2012	2004–2009
**Study design**	Retrospective	Retrospective	Prospective	Retrospective	Prospective	Retrospective	Prospective	Prospective
	Single-center	Multi-center	Multi-center	Single-center	Single-center	Multi-center	Multi-center	Single-center
**Disease**	Newly-diagnosed AML	AML/MDS	Newly-diagnosed AML	Newly-diagnosed AL	Newly-diagnosed AML	All HMs	Newly-diagnosed AML	Newly-diagnosed AML
**Patient number**	152	216	224	231	254	497	881	298
		(573 C/T courses)				(for AML)		
**Systemic antifungal prophylaxis**	Fluconazole	Fluconazole	Not remarked	No	No	Various	Posaconazole	No
	Posaconazole	Itraconazole					Fluconazole	
	Voriconazole	Posaconazole					Itaconazole	
	Echinocandins	Voriconazole					Others	
**Chemotherapy regimens**	Induction, various regimens	Induction, consolidation	Fludarabine-based induction	Standard induction	Standard induction	Various	Various	Induction, various regimens
**IFI incidence**								
**All pathogen**	13.8%^@^	5.1%^@^	4%^@^(induction)	5.9%^@^ (30 days)	48.4%^&^	3.0%^@^ (for AML)	8.7%^@^	10.7%^@^
	22.3%^&^	12.5%^&^	2%^@^(consolidation)	11.1%^@^ (100 days)			24.2%^&^	34.6%^&^
***Candida***					5.5%		2.6%	4.4%
**Mold**					42.5%		21.6%	30.2%
**Mortality** ^**%**^								
**All-cause**	26.5%			42%	23.7% (6 months)			
**IFI-attributed**			60%(induction)			22.2% (for all)		19.4%
			80%(consolidation)					

^$^only patients whose age was between 15 and 65 y/o enrolled

^@^ Including only proven or probable IFIs

^&^Including possible, probable and proven IFIs

^%^ for patients with invasive fungal infections

AmB, amphotericin B; AL, acute leucemia; AML, acute leucemia; C/T, chemotherapy; HM, hematological malignancy; IC, invasive candidiasis; IFI, invasive fungal infection; IMI, invasive mold infection; Lip-AmB, liposomal amphotericin B; MDS, myelodysplastic syndrome

The epidemiological characteristics of IFI continue to evolve in leukemia patients. A major contributor is the widespread use of azole prophylaxis since early 1990s, which results in less candidiasis but in more frequent mold infections in hematologic malignancies[20–22]. However, in our study, *Candida* spp. still predominated in culture- or histological-proven pathogens, almost twice as common as *Aspergillus* spp. (Table 2). The reason of our reversed distribution of *Candida* spp. and *Aspergillus* spp. is apparently due to the absence of routine anti-yeast azole prophylaxis administered in our study. *C*. *glabrata* and *C*. *krusei* were therefore not isolated in our study, as reported in some earlier cohorts before the launch of new azoles[5,21,23]. However, non-albicans *Candida* spp. (*C*. *tropicalis* and *C*. *parapsilosis*) were almost as common as *C*. *albicans*. This distribution is significantly different from other epidemiological analyses of invasive candidiasis from Taiwan[24,25]. However, another recently reported study showed similar findings and stated that neutropenia is correlated with non-*albican Candida* infections[26]. More information is required to confirm if the finding is true.

The lung was the most commonly involved site for IFIs. Pagano et al. reported that lung accounted for over 80% of IFI involved sites [3] and Kurosawa et al. found an incidence of 55.3% for IFIs involving the lung[9]. In our study, 75.4% IFIs were primarily localized in the lower respiratory tracts, followed by blood stream infection (9.4%), and also hepatosplenic microabscess (6.6%). The incidence of hepatosplenic fungal infection is compatible with the study in our hospital from an earlier study cohort (1995 to 2002) [27].

Induction chemotherapy represents a period with high risk of IFIs when treating patients with acute leukemia[2]. We have further postulated more risk factors related to IFIs during this study, including standard induction chemotherapy for proven/probable IFIs, and standard induction chemotherapy, age younger than 40 or older than 60 years and a poor response to chemotherapy for all-category IFIs. Anyway, only a few articles were dedicated to elucidate the risk factors of IFIs during induction. Neofytos et al.[11] identified that mucositis and baseline organ dysfunctions are important risk factors for invasive candidiasis during induction, and male gender is the only risk factor for mold infection. Hammond et al.[8] also reported male gender and persistent leukemia as risk factors for IFIs. These results indicate the heterogeneity of the study populations and treatment protocols in different reports. More investigation using large cohorts with uniformed protocols should be done to clarify this issue.

The prognosis of patients with all-category IFIs after induction chemotherapy is poor, and 19.4% IFI-attributed early mortality rate is noted for these patients. The presence of IFIs is also a significant adverse prognostic factor for newly-diagnosed non-M3 AML patients receiving induction chemotherapy (Fig 1A), especially in those receiving standard induction (Fig 1B). Furthermore, no matter when the patients are diagnosed to have possible, probable or proven IFI, their survival is uniformly worse than those without IFIs (Fig 1D). Even after multi-variate analysis, the presence of IFIs is still an independent poor prognostic indicator (Table 5). Our subgroup analysis also showed that the survival differences between patients with or without IFIs is mostly apparent in patients with partial response to treatment (log rank test *p* value = 0.02, data not shown). Furthermore, in patients who can survive for more than 3 months, escaping early IFIs insults after chemotherapy, a negative survival impact can still be observed for the presence of IFIs in induction (Fig 2). All these data suggest that not only do IFIs result in early mortality after induction chemotherapy, but they also can have long-term impacts on survival, probably through delaying chemotherapy recurring in later treatments. Surprisingly, there is only limited discussion in the literature about IFIs as a short-term or long-term prognostic factor. Girmenia et al.[28] and Michallet et al.[29] both reported that invasive fungal diseases during induction chemotherapy is an independent risk factor for overall survival in AML. Furthermore, Even et al.[30] observed that patients with fungal diseases will have higher chances to undergo significant changes to their chemotherapy, and therefore result in less event-free and overall survival even though they initially survived.

With increased concerns about the high incidences of IFIs in acute leukemia patients receiving chemotherapy, many physicians have tried to apply prophylactic agents in this immune-compromised population since the 1990s. Itraconazole and fluconazole were first found to be beneficial in studies[31,32]. In 2007, a large randomized clinical trial demonstrated the efficacy of posaconazole to prevent IFIs in AML and myelodysplastic syndrome in patients receiving induction chemotherapy[33]. With the complexity of prognostic variables in AML and the heterogeneity in treatment, our study has still demonstrated that the incidences of IFIs is high and that IFIs negatively impact patient survival. These data can provide important “real-world” insights to apply anti-fungal prophylaxis in acute leukemia patients receiving induction chemotherapy. Furthermore, based on the information provided in this study, a detailed pharmaco-economic evaluation for anti-fungal prophylaxis treatments can also be conducted to define their effectiveness.

In conclusion, the incidences of IFIs are high in acute leukemia patients who have received induction chemotherapy in Taiwan, and the short-term and long-term outcome of patients are both negatively influenced if IFIs develop in these patients. Physician should be aware of the risks and also potential risk factors so that optimal actions will be taken to ensure the best chances of cure and survival in patients with hematological malignancies.
